# Are Age Differences Between Partners Related To Gender and Generations Among Middle-Aged And Older Asian Americans?

**DOI:** 10.1093/geroni/igab046.3169

**Published:** 2021-12-17

**Authors:** Minzhi Ye, Di Mei

**Affiliations:** 1 Benjamin Rose Institute on Aging/Case Western Reserve University, Benjamin Rose Institue on Aging, Ohio, United States; 2 The University of Hong Kong, The University of Hong Kong, Hong Kong

## Abstract

Previous research has shown that women who immigrate to the United States tend to partner with much older spouses. However, most studies have focused on young people and first-generations. Spousal age differences among older Asian Americans with different generations have not been well studied. Using data from the Annual Social and Economic Supplement survey (2013-2019), we employed the segmented assimilation theory to test 7,064 married middle-aged and older (50+) Asian Americans. Logistic regression analyses were conducted to understand the association between spousal age differences and individual background, including gender, ethnicity, socioeconomic status, generation, and marriage types. Of the 3,342 men, 20% were married to wives at least 6 years younger and 2% were married to wives at least 6 years older. Men who were Japanese or had inter-ethnical marriages were more likely to marry women at least 6 years older. Men who were Indian, Vietnamese, or having an interracial marriage were more likely to marry women at least 6 years younger. Of the 3,722 women, 3% were married to husbands at least six years younger and 19% were married to husbands at least six years older. Women who were Indian or Vietnamese were more likely to marry men at least six years older. Women who had a high school diploma or were third-plus generation were more likely to marry men at least 6 years younger. The findings reflect the complexity of Asian senior marriage and provide insight for policymakers to design new or improved social integration programs for senior immigrants.

